# Deep Learning-Based Stroke Disease Prediction System Using Real-Time Bio Signals

**DOI:** 10.3390/s21134269

**Published:** 2021-06-22

**Authors:** Yoon-A Choi, Se-Jin Park, Jong-Arm Jun, Cheol-Sig Pyo, Kang-Hee Cho, Han-Sung Lee, Jae-Hak Yu

**Affiliations:** 1 KEPCO Research Institute, Korea Electric Power Corporation, 105 Munji-ro Yuseong-gu, Daejeon 34056, Korea; yoona_choi@kepco.co.kr; 2 Research Team for Health & Safety Convergence, Korea Research Institute of Standards and Science (KRISS), Daejeon 34113, Korea; sjpark@kriss.re.kr; 3 Department of KSB Convergence Research, Electronics and Telecommunications Research Institute (ETRI), Daejeon 34129, Korea; jajun@etri.re.kr (J.-A.J.); cspyo@etri.re.kr (C.-S.P.); 4 Department of Rehabilitation Medicine, Chungnam National University College of Medicine, 266 Munhwa-ro Jung-gu, Daejeon 35015, Korea; khcho@cnu.ac.kr; 5 School of Creative Convergence, Andong National University, 1375 Gyeongdong-ro (Songcheon-dong), Andong, Gyeongsangbuk-do 36729, Korea

**Keywords:** electroencephalography (EEG), stroke prediction, stroke disease analysis, deep learning, long short-term memory (LSTM), convolutional neural network (CNN), bidirectional, ensemble

## Abstract

The emergence of an aging society is inevitable due to the continued increases in life expectancy and decreases in birth rate. These social changes require new smart healthcare services for use in daily life, and COVID-19 has also led to a contactless trend necessitating more non-face-to-face health services. Due to the improvements that have been achieved in healthcare technologies, an increasing number of studies have attempted to predict and analyze certain diseases in advance. Research on stroke diseases is actively underway, particularly with the aging population. Stroke, which is fatal to the elderly, is a disease that requires continuous medical observation and monitoring, as its recurrence rate and mortality rate are very high. Most studies examining stroke disease to date have used MRI or CT images for simple classification. This clinical approach (imaging) is expensive and time-consuming while requiring bulky equipment. Recently, there has been increasing interest in using non-invasive measurable EEGs to compensate for these shortcomings. However, the prediction algorithms and processing procedures are both time-consuming because the raw data needs to be separated before the specific attributes can be obtained. Therefore, in this paper, we propose a new methodology that allows for the immediate application of deep learning models on raw EEG data without using the frequency properties of EEG. This proposed deep learning-based stroke disease prediction model was developed and trained with data collected from real-time EEG sensors. We implemented and compared different deep-learning models (LSTM, Bidirectional LSTM, CNN-LSTM, and CNN-Bidirectional LSTM) that are specialized in time series data classification and prediction. The experimental results confirmed that the raw EEG data, when wielded by the CNN-bidirectional LSTM model, can predict stroke with 94.0% accuracy with low FPR (6.0%) and FNR (5.7%), thus showing high confidence in our system. These experimental results demonstrate the feasibility of non-invasive methods that can easily measure brain waves alone to predict and monitor stroke diseases in real time during daily life. These findings are expected to lead to significant improvements for early stroke detection with reduced cost and discomfort compared to other measuring techniques.

## 1. Introduction

Stroke is a condition involving abnormalities in the brain blood vessels that result in dysfunction in certain brain locations [1]. According to a 2016 report by the World Health Organization (WHO), stroke is the second most common global cause of death in the world and the third most common global cause of disability [2]. The incidence of stroke in developing countries has more than doubled over the past 40 years [3]. Since a suitable treatment for stroke has yet to be found, early detection is paramount. CT and MRI techniques are the most common detection methods for stroke disease. However, CT and MRI are expensive and may not be suitable for developing countries or low-income earners. With stroke disease emerging as an important disease worldwide, particularly among low-income and elderly people, healthcare services desperately need a solution to help them accurately and quickly detect stroke diseases at a low cost.

Studies on the early detection and prediction of stroke are actively underway. The 2019 Global Burden Disease (GBD) study estimated cardiovascular disease incidence and patient mortality from 204 countries and regions from 1990 to 2019 [4]. According to that report, the number of deaths from cardiovascular disease in 2019 accounted for one-third of all deaths. While the death toll according to cardiovascular index rose from 11.1 million in 1990 to 18.6 million in 2019, many of the causes from cardiovascular disease were attributed to ischemic stroke. Hier et al. also reported that ischemic stroke had a very high recurrence rate of 14.1% within two years [5]. Further, recent studies have shown a link between COVID-19 and stroke, which is expected to increase the number of stroke deaths [6,7]. Kummer et al. [6] informs us that patients admitted with COVID-19 who had a stroke history were much more likely to die than those without a stroke history, while Zhang et al. [7] reported that patients with a stroke prognosis had a higher incidence of severe pneumonia and subsequent mortality according to Cox regression. Aside from these prior studies, there is still a significant lack of understanding between experimental data and data collected in real-time for stroke.

Stroke disease can be identified using blood tests; brain imaging such as CT, MRI, and X-ray; ECG and EEG; and neurological physiological methods such as induced potential tests [8]. Among these techniques, CT and MRI are most often used to determine stroke, but these involve risks such as exposure to radiation or potential allergic reactions to the contrast agents used. These tools can also be inconvenient as they involve confined spaces and require constant monitoring while also incurring separate medical costs for each examination, all of which increase the difficulty of diagnosis. New wearable electrodes provide an opportunity to measure EEG in the comfort of a participant’s home. These electrodes are attached to the head and measure the activity of brain nerve cells in a more natural setting. Brain signals are also recorded during the different sleep states, thereby allowing for pain-free and rapid examination. EEG data can be contaminated by the patient’s movement as well as environmental noises. However, it is possible to collect and test EEG data in real time and in a low-cost manner with fewer side effects than the aforementioned imaging techniques. Due to these advantages, 24-h EEG measurements are considered a useful, low-cost method for monitoring stroke disease with high recurrence rates in daily life.

According to the literature review, a number of studies have analyzed diseases such as seizure, Alzheimer’s, and stroke using EEG, while other studies have also correlated the level of sleep and emotions using EEG testing [9,10,11,12,13]. However, most of these studies focused on simple classifications or used preset frequency features extracted for experimentation. Thus, additional time and cost are needed to separate raw data into frequency domains, meaning that real-time health monitoring is not currently feasible with the technology that has been reported to date. A recent study was able to classify seizure patients using raw EEG data alone, representing a promising step toward real-time health monitoring using EEG [14,15]. Therefore, in this paper, we propose a real-time stroke monitoring system to predict the degree of risk of stroke in real time by collecting raw EEG data.

Using real-time EEG data, our system can predict stroke diseases in elderly Koreans. To test this system, we developed a walking protocol that reflects the everyday life of an elderly Korean. The EEG data used in this work were measured and collected from Korean seniors aged 65 or older. To compare between deep learning models and machine learning models, the collected EEG data were separated into raw data and frequency domain-extracted data, respectively. Each set of raw EEG data from six channels (Fz, T7, C1, C2, T8, and Oz) undergoes a fast Fourier transform (FFT), and 66 values in total were extracted and used in the experiment. The initial results show that the raw data alone could be used to predict stroke disease with high accuracy. To determine which deep learning model is the most suitable for real-time EEG data, a comparison of the predictive accuracies of LSTM, bidirectional LSTM, CNN-LSTM, and CNN-bidirectional LSTM models was conducted. These algorithms were chosen because they are known to be suitable for real-time data learning based on the characteristics of EEG data. Our experiments showed 94.0% accuracy for CNN-Bidirectional LSTM models with very low false negative rate (FNR) and false positive rate (FPR) at 5.7% and 6.0%, respectively, confirming high confidence in the results. Meanwhile, using power value, we showed 81.4% accuracy along with 18.5% FPR and 17.3% FNR in CNN-bidirectional LSTM models. Further, experiments using relative value showed 89.2% accuracy as well as FPR and FNR of 12.5% and 8.4%, respectively, in CNN-LSTM models. These experimental results indicate that raw EEG data alone can be used to accurately detect and predict stroke diseases without separating the frequency attribute values. We also confirmed that the system proposed in this paper accurately predicts the precursor symptoms of stroke disease with very high mortality and recurrence rates in real time, and thus represents a low-cost method that enables health monitoring during the daily lives of the elderly.

The paper is organized as follows. Section 2 examines prior research involved in EEG features in stroke patients as well as computer engineering studies related to stroke prediction. Section 3 explores deep learning-based stroke disease prediction systems with real-time brainwave data proposed in the paper, and also discusses prediction methodologies using raw data and frequency properties of brainwaves. Section 4 discusses the real-time stroke disease prediction experiments conducted in this study and the analysis results with deep learning models specialized in time series signal data prediction, while Chapter 5 concludes and proposes future study directions.

## 2. Related Works

### 2.1. Related Work on Stroke EEG

Wijaya et al. [16] proposed a method for detecting ischemic stroke disease through various signal process and machine learning techniques. They were able to accurately classify 40 stroke patients and 40 normal people using EEG data from six channels. The data underwent various types of signal processing such as fast Fourier transform (FFT), wavelet transform (WT), short-time Fourier transform (SFFT), and power spectral density (PSD) techniques before multiple frequency domains data were subjected to a multi-layer perceptron (MLP) and decision tree techniques for the detection of ischemic stroke. The proposed system had a test accuracy of 95%. Rahma et al. [17] used an extreme Llearning machine (ELM) to automatically classify Athens Insomnia Scale (AIS) severity based on EEG signals through digital signal processing such as feedforward and WT structures. Their algorithm confirms the usefulness of EEG in signal-based AIS classification with more than 72% test accuracy, sensitivity, and specificity according to the National Institutes of Health Stroke Scale (NIHSS). Li et al. [18] et al. introduced various machine learning methodologies for stroke risk level classification in Chinese stroke screening and presented the experimental results. In particular, the experimental results were analyzed with more than 8 machine learning algorithms, including the logistic regression model. In the experiment using boosting decision tree, the results of recall and precision were 99.94% and 97.33%, respectively. Li et al. suggested an improvement plan for the screening method program that is actually used up to now. ELM was also used by Adhi et al. [19] via detrended fluctuation analysis (DFA) for the automatic detection of ischemic stroke and normal controls. Signal processing was performed on 18 channels, and the subject’s scale index was used as an input to the ELM to classify ischemic stroke. These experiments used 120 hidden neurons and sines as an activation function of EML and obtained 84% accuracy, 87% specificity, and 82% sensitivity. Djamal et al. [20] proposed a method for identifying stroke patients after the occurrence of stroke using a convolutional neural network (CNN). They used wavelets to extract brainwave signal information for use as a feature in machine learning that reflects the patient’s condition after stroke. The extracted features are alpha, beta, theta, delta, and mu waves of brain waves, and the accuracy of the test data was 90% with amplitude and beta features while it was 70% without them.

Studies that analyze EEG characteristics in stroke patients have shown that there are distinct characteristics in three frequency ranges: alpha wave (8~13 Hz), theta wave (4~8 Hz), and delta wave (1~4 Hz). Schneider et al. [21] analyzed brain wave frequency and brain wave topography in 20 mild stroke patients. Their results confirmed a meaningful decrease in alpha wave and increase in delta wave activity in 17 mild stroke patients. In another study, Finnigan et al. [22] reported that abnormal waveforms in the alpha and delta wave were found in stroke patients. Specifically, in stroke patients, the delta wave showed abnormal and slower characteristics than that in normal people, while the alpha wave showed reduced normal and fast activity. This information can be used to detect brain waves in stroke patients using the values of delta, delta and alpha power ratio (DAR), and power ratio index (PRI). Varelas et al. [23] reported that a rhythmic and large amplitude sum of theta and delta power values appeared in stroke patients with additional epilepsy. Ip et al. [24] confirmed the occurrence of changes in theta/delta values in stroke patients, as well as a sharp increase in the values of delta (δ), alpha (α), beta (β) (14~30 Hz), and high gamma (γ) (over 30 Hz) in the right brain hemisphere after stroke. Kim et al. [25] conducted a follow-up and made behavioral observations for three minutes in 12 stroke patients and a normal control group.

### 2.2. Related Work on AI-based Stroke Classification

Currently, many deep learning-based studies use CT or MRI images to detect stroke [26,27,28,29,30,31,32]. For example, in a study classifying hemorrhagic stroke and ischemic stroke using brain CT images, Gautam et al. [26] achieved a classifier performance of up to 98.77%. Kalchbrenner et al. demonstrated that their proposed 13-layer CNN [27] model showed better performance in comparative experiments with AlexNet [28] and ResNET50 [29]. Chin et al. [30] applied data augmentation to CT images of ischemic stroke patients to expand the number of patch images, used them as input to CNN models to detect ischemic stroke, and obtained more than 90% accuracy. Liu et al. [31] proposed a Res-CNN model that automatically classifies acute ischemic stroke in MRIs. The Res-CNN model solved the performance degradation problem using the residual unit, and it improved the model performance through data expansion. Dourado et al. [32] presented a CT image classification IoT framework applied with a CNN to classify ischemic stroke and hemorrhagic stroke. In that same framework, they further experimented with machine learning methods like Bayesian [33], MLP [34], k-nearest neighbor [35], random forest [36], and support vector machines (SVM) [37] by applying the various machine learning concept [38], and they validated the model with 100% accuracy. They reported that training and testing times of 0.015 s and 0.001 s, respectively, when using the Bayesian Classifier, can be classified as ischemic stroke and hemorrhagic stroke with high accuracy in a short time.

Beyond images, various biological signals have also been used to predict stroke diseases. For example, Yu et al. [39] proposed a pre-detection and prediction method for machine learning and deep learning-based stroke diseases that measure the electrical activities of thighs and calves with EMG biological signal sensors, which can easily be used to acquire data during daily activities. They experimentally verified an accuracy of more than 90% using real-time collected data. Through these experiments, that study demonstrated a novel method that can verify stroke disease with high accuracy based on the pre-symptoms of stroke, body falling, and the degrees of both leg muscles. In another study, Xie et al. [40] proposed CNN-based DenseNet for stroke disease classification and prediction based on ECG data collected using 12 leads, and they obtained 99.99% training accuracy and 85.82% testing accuracy using fine-tuned models for the correlation between stroke and ECG. However, they used other biological signals that are not closely related to the brain. Thus, there is a need for studies using brain waves with AI.

Fawaz et al. [41] conducted a study to expand data using techniques such as conditional general adversarial network (CGAN) [42] to address the lack of data in stroke patients, and to classify EEG in stroke patients through the LSTM encoding process and the frequency mapping phase for loss functions. The proposed model uses frequency properties, and its performance is compared with those of the typical deep learning models of feed forward [43], CNN [27,28], bidirectional LSTM [44], and CNN-bidirectional LSTM [45] as baseline models. The model proposed in that study achieved a high accuracy of 90.5% for classifying stroke patients. In a different study, Guntari et al. [46] classified poststroke EEG signals using RNN and genetic algorithms (GA). The stroke data used in Zhang et al. were extracted using wavelet [47], and the initial 215,040 data points were reduced to 29,160 on 14 channels containing power values; this reduced the figures time from 80 s to 50 s, and an experimental accuracy of 90.0% was obtained. Giri et al. [48] conducted a stroke identification study based on EEG signals and electrooculography (EOG) signals using 1D CNN and batch normalization. The collected data consisted of the relative values, correlation aspects, variance, spectral mean, entropy, kurtosis, and fractal indices of brain waves through feature extraction steps, and an F-Score of 86.1% was ultimately obtained.

## 3. Deep Learning-Based Stroke Disease Prediction System Using EEG

**Figure 1:**
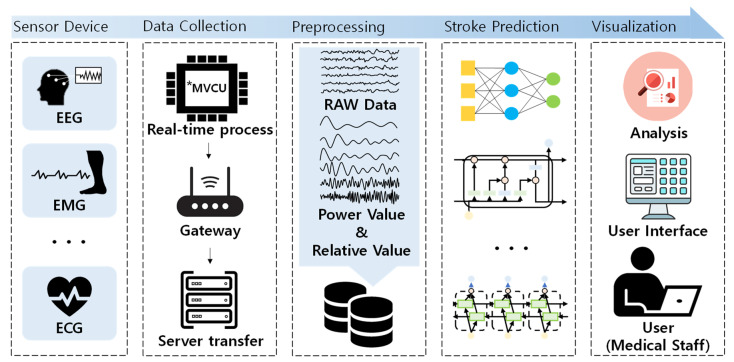
Elderly stroke monitoring system based on deep learning using bio signals (* MVCU: multi vital-signals collector units).

We propose a novel system for predicting stroke based on deep learning using the raw and attribute values of EEG collected in real time, as presented in Figure 1. The proposed system is composed of (1) a module that collects data in real time; (2) a module that transmits the real-time generated biological signals to the server; (3) a module that analyzes the stored biological data, then extracts and manages frequency attributes; (4) a deep learning-based learning and prediction module; and (5) a biological-signal-based stroke prediction analysis. Next, the initial system extracts and stores a set of pretreatment processes and features based on various biological signal data, such as EEG data and ECG data collected from elderly people. Finally, the stored bio-signal data is used as input for deep learning models to predict and analyze stroke anomalies in older adults in real time.

### 3.1. Sensor Device and Data Collection Module

We measured and collected various bio-signal data, such as electroencephalography (EEG), electrocardiography (ECG), electromyography (EMG), motion, etc., for the classification and prediction of stroke diseases using bio-signals. Although all bio-signal data were collected in real time, we only used the EEG data to validate the stroke disease prediction and analysis. There are two main methods of measuring brain wave data. In the first method, scalp EEG can be measured painlessly by attaching electrodes to the outside of the skull, i.e., to the scalp. In the second method, electrodes can be surgically inserted into the subject’s cerebral cortex. This method provides a relatively neat signal, but it is highly invasive and involves the risk of incision into the skull. In this paper, the data are acquired using non-invasive scalp EEGs to allow for easier data collection during daily activities and verification accuracy for stroke prediction.

### 3.2. Data Preprocessing Module

Once the device is set, the EEG data are collected at a sampling rate of 1000 Hz from six channels, and the frequency attribute values are extracted from the raw. The extracted frequency attribute values are categorized into two major values: (1) the power value indicating the extent to which each frequency component has appeared and (2) the relative value indicating the relative proportion of each component in the frequency domain for the entire region. Basic EEG biological tests use power values, which may give different amplitude sizes of EEG waveforms due to individual differences in scalp and skull thickness as well as the contact with the electrode and skin. Therefore, for experiments with multiple subjects, it is desirable to use the relative value of each region for analysis due to the large individual deviation of the power value. The relative values of the frequency domain are extracted for comparison with the results of the experiments using raw data. The frequency attribute value extraction extracts values such as alpha, beta, etc. of the power value by applying FFT to raw EEG data. Additional functions were performed to disaggregate the frequency range based on the extracted values, or a ratio of each value was applied to extract a total of 66 attribute values. In addition, based on the power value, we further extract the relative value representing the relative ratio and design it for use in experiments.

**Figure 2:**
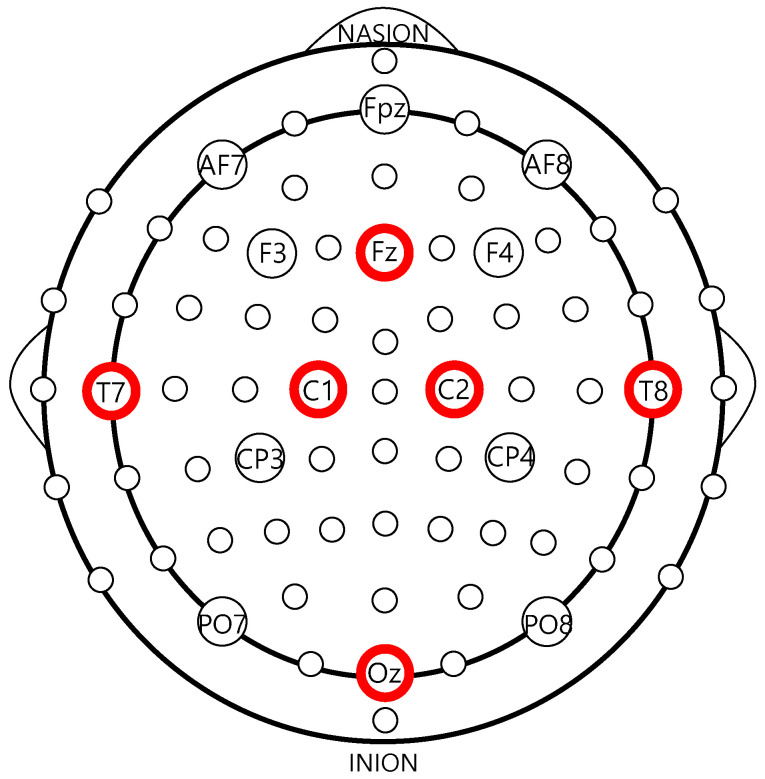
Six-channel measurement and collection locations of EEG vital-signals.

**Figure 3:**
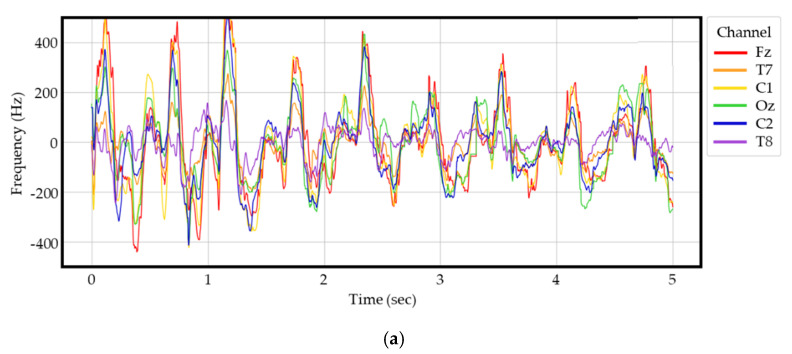

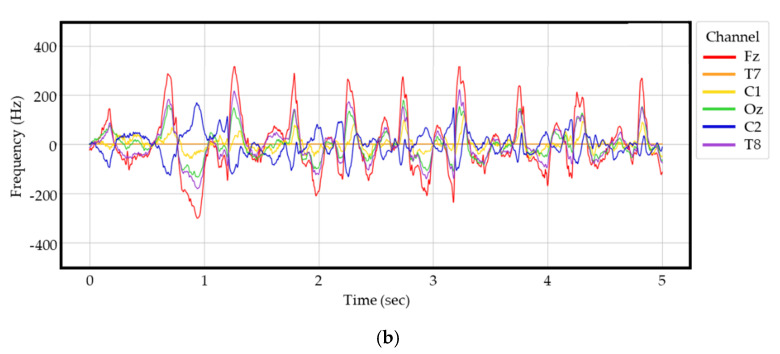
Raw EEG signal samples: (**a**) Raw EEG signals from elderly stroke patients; (**b**) Raw EEG signal samples from control group.

The lines on the graph represent the raw values collected from the six channels shown in Figure 2, while the vertical axis represents frequency in Hz and the horizontal axis represents time. For time, periods of at least five seconds were used to differentiate the walking data. Overall, using only raw EEG data from stroke patients and normal controls to distinguish and determine stroke disease can be difficult for medical professionals. In addition, medical professionals need time to make a proper diagnosis of stroke. In this study, we conduct experiments and validations with the goal of providing meaningful information and ultimately designing and implementing systems that can help medical staff make judgments more quickly, rather than simply attempt to develop a system that can predict stroke early. Figure 3 shows examples of the raw EEG data in (a) stroke patients and (b) normal controls.

### 3.3. Stroke Prediction Module

**Figure 4:**
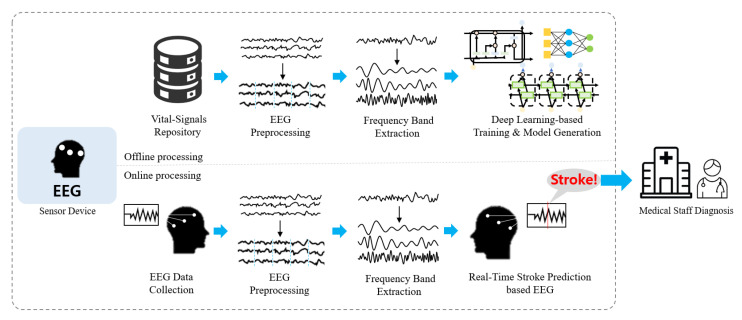
Stroke prediction module structure based on deep learning.

The stroke prediction module for the elderly using deep learning-based real-time EEG data proposed in this paper consists of two units, as illustrated in Figure 4. For the offline processing unit, the EEG data are extracted from a database storing the data on various biological signals such as EEG, ECG, and EMG, and a series of processes are followed to learn and tune the parameters based on deep learning. The online processing unit analyzes brainwave data collected in real time based on features learned from the offline processing unit and delivers information about stroke prediction to medical staff in real time. This information can then be communicated to help medical staff predict stroke faster and make accurate diagnoses.

**Figure 5:**
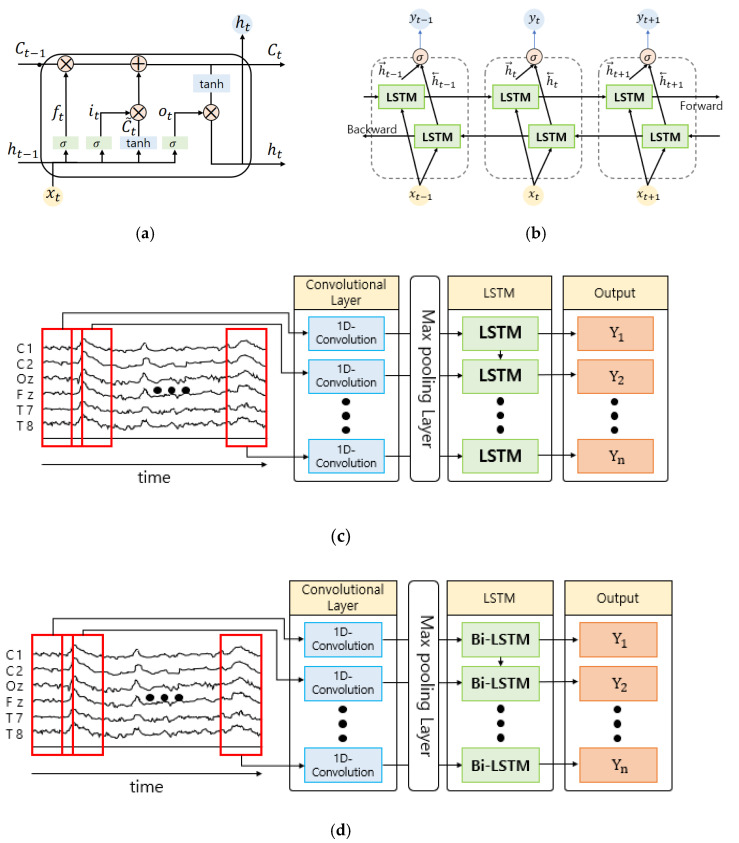
The architecture of the four deep learning models used in the experiment: (**a**) LSTM; (**b**) Bidirectional LSTM; (**c**) CNN–LSTM; (**d**) CNN-Bidirectional LSTM.

EEG data are time series data that consist of sequential values over time, and it is necessary to consider time information in the learning process. Therefore, in our stroke prediction module we transform and optimize the structure of a deep learning model to obtain a model that can address the characteristics of time series data well and use it in experiments. The deep learning models that were analyzed were CNN [27,28], LSTM [49], bidirectional LSTM [44], etc. CNNs can reduce computation volume through the use of basically shared parameters, and they have the advantage of mitigating overfitting. Moreover, CNNs are increasingly being used to research and develop models by extracting the optimal properties to improve classification and prediction performance. The LSTM model can predict the next stage of time series data by considering both future and historical information of time series data, which can solve the long-term dependency problem. Finally, Bidirectional LSTM is a structure that can overcome the structural limitations of LSTM, where the results of the latter tend to converge based on the last-minute pattern by adding a reverse processing layer to the existing LSTM layer. In this work, we selected four models to fit the time series data classification using the advantages of each model, which are illustrated in Figure 5.

#### 3.3.1. Long-Short Term Memory (LSTM)

The existing RNN is a kind of ANN wherein information persists inside the neural network, and it has been used for image caption generation, automatic translation, etc. [50]. However, RNN has the disadvantage of its learning ability gradually decreasing due to the reduction in its slope when the distance between information grows, i.e., when the length of the input sequence increases. To address these problems, we use the LSTM [49] model, which adds a cell-state to the hidden-state of the RNN. LSTM consists of a forget gate, an input gate, and an output gate. The forget gate $f_{t}$ is a gate for forgetting past information inside a neural network, which preserves the information entirely if the formula result is 1 and discards the information if it is 0.

(1) $$f_{t}=\sigma \left(W_{f}\cdot \left[h_{t},\;x_{t}\right]+b_{f}\right)$$

The input gate $i_{t}$ is a gate for remembering the current information inside a neural network, which determines which new information is stored in the cell state. First, Equation (2) determines which value to update, then prepares to add the new candidate values, the ${\tilde{C}}_{t}$ vector, to the cell state through Equation (3). The state can be said to be prepared by combining the two sets of information from Equations (2) and (3).

(2) $$i_{t}=\sigma \left(W_{i}\cdot \left[h_{t-1},\;x_{t}\right]+b_{i}\right)$$

(3) $${\tilde{C}}_{t}=\tan h\left(W_{C}\cdot \left[h_{t-1},\;x_{t}\right]+b_{C}\right)$$

It then updates the past state, $C_{t-1}$, to create a new cell state, $C_{t}$. As shown in Equation (4), the previous state is multiplied by $f_{t}$, while forgetting what one has chosen to forget at the very first step and adding $i_{t}*{\tilde{C}}_{t}$. $i_{t}*{\tilde{C}}_{t}$ is a value that has been increased or decreased by how much the value was supposed to be updated.

(4) $$C_{t}=f_{t}*C_{t-1}+i_{t}*{\tilde{C}}_{t}$$

Finally, the output gate $o_{t}$ determines how much of the information is represented inside the neural network, where output is filtered based on the cell state. The cell state is input into the tanh layer to receive a value between −1 and 1. This value is multiplied by the output of the previously calculated sigmoid gate, which allows only the desired part to be expressed as output. This process is as shown in Equations (5) and (6).

(5) $$o_{t}=\sigma (W_{o}\left[h_{t-1},\;x_{t}\right]+b_{o}$$

(6) $$h_{t}=o_{t}*\tan h\left(C_{t}\right)$$

#### 3.3.2. Bidirectional LSTM

Bidirectional RNN (BRNN) is a model used when time series data exhibit significant results in forward inference from the past to the future, as well as reverse inference from future to past [51], i.e., it is used to predict labels for current data through past sequences and future sequences. For this model, there are hidden layers with information on the forward states and hidden layers with information on the backwards states, and these two layers are not interconnected. However, the input value is passed to both hidden layers, and the output layer also receives the value from the two hidden layers to calculate the final output. This is equivalent to Equations (7)–(9), which calculate the activation output $\vec{h_{t}}$ of the forward hidden layer, the activation output $\overset{\leftarrow }{h_{t}}$ of the background hidden layer, and the output $y_{t}$ of the output layer at time t.

(7) $$\vec{h_{t}}=\sigma (W_{x\vec{h}}x_{t}+W_{\vec{h}\vec{h}}{\vec{h}}_{t-1}+b_{\vec{h}}$$

(8) $$\overset{\leftarrow }{h_{t}},=\sigma (W_{x\overset{\leftarrow }{h}}x_{t}+W_{\overset{\leftarrow }{h}\overset{\leftarrow }{h}}{\overset{\leftarrow }{h}}_{t+1}+b_{\overset{\leftarrow }{h}}$$

(9) $$y_{t}=W_{\vec{h}y}{\vec{h}}_{t}+W_{\overset{\leftarrow }{h}y}{\overset{\leftarrow }{h}}_{t}+b_{y}$$

We use bidirectional LSTM (BiLSTM) [44] for the experiments in this paper by applying the LSTM network instead of RNN in bidirectional RNN.

#### 3.3.3. CNN–LSTM

CNN [27,28] is capable of implementing complex nonlinear models, and it is specialized in image and speech recognition. While LSTM models perform well on sophisticated time series data that follow a particular trend, for data that do not exhibit a particular trend and that show severe changes, the prediction value converges upon a specific constant value, thus resulting in poor accuracy. To solve this problem, we combine CNNs that have the advantage of extracting the characteristics of time series data and LSTM models that predict the time series data for the next step by considering both historical and future information regarding time series data [52]. The time series data entered can be more accurately classified and predicted because it extracts the properties of the data through the CNN layer, then passes through the LSTM layer that exploits past and future information.

#### 3.3.4. CNN-Bidirectional LSTM

As mentioned earlier, we combine CNN models at the front of the LSTM model to compensate for the shortcomings of LSTM [49]. We also combine a bidirectional LSTM model with a CNN that can simultaneously make reverse predictions from the future to the past direction and with the underlying forward LSTM model that makes predictions from the past to the future [45].

## 4. Experiments and Result Analysis

### 4.1. Data Collection and Description

As explained in Section 3.1, from the various biological signals collected, the experiments were conducted using only EEG data. The measurement and collection of EEG data were conducted at the emergency medical center of Chungnam National University Hospital for senior citizens aged 65 or older from 2017 to 2018. The selected subjects were patients who had received rehabilitation treatment for stroke and who had been diagnosed with stroke within the first month. The experiment involved 48 stroke patients and 75 normal patients from 2017, as well as 13 stroke patients and 137 normal patients from 2018. To achieve an equal comparison, 61 stroke patients and 61 randomly selected control data were ultimately selected for analysis. Five specific daily activity protocols were implemented, including walking, chair sitting and standing, standing, moving objects, and sleeping. After being equipped with the vital-signal collection sensors, all subjects were trained once before the measurement protocol. The first and last of the five measurement protocols were excluded from the experimental data, as these data were likely to reflect the subject’s tension, discomfort, and fatigue. Of the total 273 subjects, 227 cases were evaluated by the medical staff as the NIHSS value for evaluating the severity of stroke. There were 117 males, with a mean age of 74.44, a standard deviation of 6.775, a maximum age of 90, and a minimum age of 65 years. There were 110 females, with an average age of 77.82, a standard deviation of 6.661, a maximum age of 99 years, and a minimum age of 65 years, just like men. Including both males and females, there were 13 elderly patients with NIHSS scores of 0, 153 patients with a score from 1 to 4, 42 patients with a score from 5 to 15, and 8 patients with a score from 21 to 42. In this paper, the patients with an NIHSS score of 5 or higher were specified as stroke patients, and this is a criterion defined by prior references that was agreed upon by the medical staff at Chungnam National University Hospital.

The raw data values acquired from the six channels were extracted using FFT techniques, as described in Section 3.2. After redefining the value of the extracted attributes, a total of 66 power values and relative values were used, as listed in Table 1.

### 4.2. Performance Evaluation and Indicators for Experiments

All experiments were performed in the same experimental environment: OS, Ubuntu 18.04.4 LTS; CPU, Intel Core i7-9800X; GPU, 2 X NVIDIA Quadro RTX 8000; and RAM, 256 MB. In this paper, we utilize statistical metrics used for disease screening to evaluate the performance of our system. The performance evaluation indicators used are defined below.

① Sensitivity: Percentage of stroke patients who have tested positive.
② Specificity: Percentage of non-stroke patients who have tested negative.
③ False Positive Rate: Percentage of non-stroke patients who have tested positive.
④ False Negative Rate: Percentage of stroke patients who have tested negative.
⑤ Accuracy: Percentage of stroke patients determined as positive and non-patients as negative.
⑥ Precision: Percentage of people who are actually stroke patients among those who have tested positive.
⑦ Recall: Percentage of stroke patients who have previously tested positive.
⑧ F1-Score (Harmonic Mean of Precision and Recall): Percentage of stroke patients who have previously tested positive.

To determine the performance of the classifiers, it is important to have a high accuracy of the model, but the sensitivity and the specificity must also be high, while the false positive and false negative ratios should be low (See Table 2 below). If a non-suitable diagnostic method is used, the sensitivity and the specificity tend to decrease when one side increases, so both should have a high value to achieve a good diagnostic method. When the false positive ratio is actually negative and the result is positive, this can lead to wrong data classification and additional cost. When the false negative ratio is actually positive and the result is negative, this can have a critical impact on the patient’s life, which is used as an important performance evaluation indicator when using medical data.

(10) $$Sensitivity\;\left(=Recall\right)=\frac{TP}{TP+FN}$$

(11) $$Specificity=\frac{TN}{FP+TN}$$

(12) False Positive Rate (FPR)=1−Specificity

(13) False Negative Rate (FNR)=1−Sensitivity

(14) $$Accuracy=\frac{TP+TN}{TP+FN+FP+TN}$$

(15) $$Precision=\frac{TP}{TP+FP}$$

(16) $$F1-Score=2\times \frac{Precision\times Recall}{Precision+Recall}$$

### 4.3. Experimental Results and Analysis

The deep learning-based EEG data stroke classification experiments in this study used LSTM, bidirectional LSTM, CNN-LSTM, and CNN-bidirectional LSTM models, as described in Section 3, and the experiments were conducted by entering three types of data (raw values, power values, and relative values) into each model. Every experiment was performed ten times, and the mean value was shown as the final result.

Table 3, Table 4, Table 5, Table 6, Table 7 and Table 8 list the results of the stroke prediction experiments performed with each model based on the type of data. The experimental results are presented in terms of accuracy, F1-Score, precision, sensitivity, specificity, FNR, and FPR using the performance evaluation metrics described in Section 4.2. Of the total data, 80% were used for learning in the experiments, and the remaining 20% were used for prediction and validation. In this experiment, a data set was constructed according to five-fold validation. We used the average value of the prediction results as a performance index to discover a more generalizable stroke disease prediction model.

**Figure 6:**
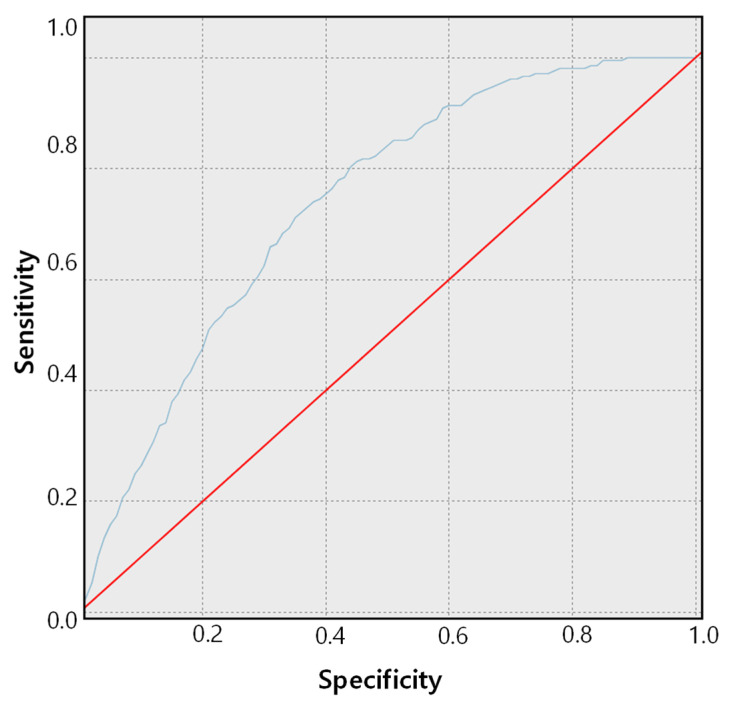
The ROC curve of the CNN-bidirectional LSTM model using raw EEG bio signals.

The ROC (receiver operating characteristic) curve of the CNN-bidirectional LSTM models in Table 4 is presented in Figure 6. The ROC curve is an index expressing the threshold and performance of binary classification prediction of stroke disease, where the *x*-axis indicates specificity and the *y*-axis indicates sensitivity.

When we conducted predictive experiments applying each deep learning model using raw values, the experimental results obtained using the CNN-bidirectional LSTM models were the highest at 94.0%, as presented in Table 3. The low false negative and false positive ratios mean that the rates of classifying stroke patients as normal and normal patients as stroke patients are both very low. CNN-bidirectional LSTM showed satisfactory experimental results for not only accuracy but also performance evaluations such as precision and F1-Score.

Table 5 and Table 6 list the results of the experiments conducted by applying each deep learning model with power values. In this experiment, we show that the CNN-bidirectional LSTM model performed best with a stroke disease prediction accuracy of 81.4%. However, the overall performance was significantly lower than that obtained using raw values. In particular, the FPR (18.5%) and FNR (17.3%) are both high, which are too low to have any clinical meaning and for medical staff to use as a stroke disease judgment.

Finally, after conducting experiments by applying each deep learning model using relative values, we experimentally confirmed that the Bidirectional LSTM model has the highest predictive accuracy at 89.2%. The performance of the bidirectional LSTM was slightly higher when using the relative value than when using the power value, but the overall performance was lower than the experimental results obtained using the raw value. To summarize, CNN-bidirectional LSTM models provide the best performance when raw data values are used. Altogether, the results show that the FPR and FNR alone can predict stroke with high performance. This means that the time needed to detect stroke disease can be shortened without needing to go through the frequency attribute extraction process. For the three data types, the hyper-parameters for each model are listed in Table 9 below.

## 5. Conclusions

In this paper, we propose a system that enables the early detection and prediction of stroke disease based on deep learning using EEG raw data, power values, and relative values. The analysis of the experimental results of raw data, power values, and relative values showed that using raw data achieved the highest stroke prediction accuracy. In addition, we confirmed that the specificity and sensitivity of the experimental results using raw data are lower than those of other data and models, with very low FPR and FNR. This is an important experimental result indicating that the early detection and onset of stroke disease can be accurately predicted using raw EEG data alone, without requiring the cumbersome process of separating frequency domain properties. The system proposed in this paper can provide useful analytical information for medical staff, patients with stroke with high recurrence potential, or elderly people with high incidence of stroke. It is a significant finding that stroke can be predicted at low cost during daily activities such as walking situations. This study is meaningful, as it can detect the risk of stroke early, before an individual is taken to the emergency room, thus allowing for access to treatment within the golden period. However, to improve the predictive accuracy and performance of real-time predictive models of stroke disease, analysis and predictive models should be studied by integrating health examination data and CT analysis information in a clinical setting.

In future research, we will study accurate predictions and in-depth interpretations of stroke disease using various biological signals such as EMG, ECG, and gait motion, as well as other EEG data. Further, we intend to research and develop systems that provide more reliable and clinically interpretable stroke prediction results by conducting multimodal studies that combine electronic medical recording (EMR) data, such as individual health checkups, with CT or MRI scans and interpretating the information.

## Figures and Tables

**Table 1:** Detailed descriptions of newly defined and extracted EEG attributes.

Frequency Band	Meaning and Description
Delta	Delta power (1~4 Hz)
Theta	Theta power (4~8 Hz)
Alpha	Alpha power (8~13 Hz)
Beta	Beta power (14~30 Hz)
Gamma	Gamma power (30 Hz or more)
Low Beta	Low beta power (12~25 Hz)
High Beta	High beta power (25~30 Hz)
Theta to Beta	The value of the beta ratio in theta (extracting abnormal theta waves)
DAR	Ratio of mean power (delta/alpha)
IDAR	Inverse ratio of DAR (alpha/delta)
PRI	Power ratio index (delta+theta to alpha+beta), Low frequency to high frequency

**Table 2:** Confusion matrix for performance evaluation.

	True	Stroke	Normal
Predicted			
**Stroke**		*TP* ^1^	*FP* ^2^
**Normal**		*FN* ^3^	*TN* ^4^

^1^ TP (True Positive): Indicator predicting stroke elderly as stroke elderly. ^2^ FP (False Positive): Indicator predicting stroke elderly as general elderly (normal). ^3^ FN (False Negative): Indicator predicting general elderly (normal) as stroke elderly. ^4^ TN (True Negative): Indicator predicting general elderly (normal) as general elderly.

**Table 3:** DL models’ accuracy, precision, and F1-score based on raw values.

	Evaluation Method	Models	Accuracy	Precision	F1-Score
Data Sets					
Raw data		LSTM	70.1	67.9	75.4
		Bidirectional LSTM	91.8	85.3	91.7
		CNN-LSTM	93.7	96.6	93.7
		CNN-Bidirectional LSTM	94.0	94.6	94.1

**Table 4:** DL models’ sensitivity, specificity, FPR, and FNR based on raw values.

	Evaluation Method	Models	Sensitivity	Specificity	FPR ^1^	FNR ^2^
Data Sets						
Raw data		LSTM	90.2	50.2	49.8	9.9
		Bidirectional LSTM	90.4	93.5	6.5	9.6
		CNN-LSTM	91.9	96.1	3.9	8.1
		CNN-Bidirectional LSTM	94.0	94.3	6.0	5.7

^1^ FPR (False Positive Rate): Indicator of the percentage of general elderly (normal) expected to be stroke elderly. ^2^ FNP (False Negative Rate): Indicator of the percentage of stroke elderly predicted as general elderly (normal).

**Table 5:** DL models’ accuracy, precision, and F1-score based on power values.

	Evaluation Method	Models	Accuracy	Precision	F1-Score
Data Sets					
Power value		LSTM	69.5	69.5	68.8
		Bidirectional LSTM	79.5	76.4	80.8
		CNN-LSTM	74.7	71.4	77.3
		CNN-Bidirectional LSTM	81.4	80.8	80.1

**Table 6:** DL models’ sensitivity, specificity, FPR, and FNR based on power values.

	Evaluation Method	Models	Sensitivity	Specificity	FPR	FNR
Data Sets						
Power value		LSTM	73.8	64.2	35.8	26.2
		Bidirectional LSTM	88.3	70.8	29.2	11.7
		CNN-LSTM	86.8	65.1	34.9	13.2
		CNN-Bidirectional LSTM	82.7	81.5	18.5	17.3

**Table 7:** DL models’ accuracy, precision, and F1-score based on relative values.

	Evaluation Method	Models	Accuracy	Precision	F1-Score
Data Sets					
Relative value		LSTM	81.0	82.8	80.7
		Bidirectional LSTM	89.2	86.9	88.8
		CNN-LSTM	84.0	82.4	83.7
		CNN-Bidirectional LSTM	86.2	87.3	85.8

**Table 8:** DL models’ sensitivity, specificity, FPR, and FNR based on relative values.

	Evaluation Method	Models	Sensitivity	Specificity	FPR	FNR
Data Sets						
Relative value		LSTM	79.3	82.5	17.5	20.7
		Bidirectional LSTM	91.6	87.5	12.5	8.4
		CNN-LSTM	85.2	87.3	12.7	14.8
		CNN-Bidirectional LSTM	86.0	83.1	17.0	14.0

**Table 9:** Hyper-parameters of each model.

Data Sets	Models	Learning Rate	Batch Size	Epoch	Optimizer
Raw	LSTM	0.01	64	50	Adam
	Bidirectional LSTM	0.001	128	100	”
	CNN- LSTM	0.01	64	200	”
	CNN-Bidirectional LSTM	0.001	64	500	”
Power	LSTM	0.0001	64	300	”
	Bidirectional LSTM	0.001	32	300	”
	CNN- LSTM	0.01	128	500	”
	CNN-Bidirectional LSTM	0.001	64	500	”
Relative	LSTM	0.0001	128	500	”
	Bidirectional LSTM	0.001	32	300	”
	CNN- LSTM	0.001	64	300	”
	CNN-Bidirectional LSTM	0.01	64	300	”
